# Tolerance of Facultative Metallophyte *Carlina acaulis* to Cadmium Relies on Chelating and Antioxidative Metabolites

**DOI:** 10.3390/ijms21082828

**Published:** 2020-04-18

**Authors:** Sławomir Dresler, Maciej Strzemski, Jozef Kováčik, Jan Sawicki, Michał Staniak, Magdalena Wójciak, Ireneusz Sowa, Barbara Hawrylak-Nowak

**Affiliations:** 1 Department of Plant Physiology and Biophysics, Institute of Biological Science, Maria Curie-Skłodowska University, Akademicka 19, 20-033 Lublin, Poland; 2 Department of Analytical Chemistry, Medical University of Lublin, Chodźki 4a, 20-093 Lublin, Poland; maciej.strzemski@poczta.onet.pl (M.S.); jan.sawicki@mgr.farm (J.S.); michal_staniak@wp.pl (M.S.); kosiorma@wp.pl (M.W.); i.sowa@umlub.pl (I.S.); 3 Department of Biology, University of Trnava, Priemyselná 4, 918 43 Trnava, Slovakia; jozkovacik@yahoo.com; 4 Department of Botany and Plant Physiology, University of Life Sciences in Lublin, Akademicka 15, 20-950 Lublin, Poland; barbara.nowak@up.lublin.pl

**Keywords:** antioxidants, glutathione, heavy metals, phenolic metabolites, terpenoids

## Abstract

The impact of long-term chronic cadmium stress (ChS, 0.1 µM Cd, 85 days) or short-term acute cadmium stress (AS, 10 µM Cd, 4 days) on *Carlina acaulis* (Asteraceae) metabolites was compared to identify specific traits. The content of Cd was higher under AS in all organs in comparison with ChS (130 vs. 16 µg·g^−1^ DW, 7.9 vs. 3.2 µg·g^−1^ DW, and 11.5 vs. 2.4 µg·g^−1^ DW in roots, leaves, and trichomes, respectively) while shoot bioaccumulation factor under ChS (ca. 280) indicates efficient Cd accumulation. High content of Cd in the trichomes from the AS treatment may be an anatomical adaptation mechanism. ChS evoked an increase in root biomass (hormesis), while the impact on shoot biomass was not significant in any treatment. The amounts of ascorbic acid and sum of phytochelatins were higher in the shoots but organic (malic and citric) acids dominated in the roots of plants from the ChS treatment. Chlorogenic acid, but not ursolic and oleanolic acids, was elevated by ChS. These data indicate that both chelation and enhancement of antioxidative power contribute to protection of plants exposed to long-term (chronic) Cd presence with subsequent hormetic effect.

## 1. Introduction

Response of plants to cadmium (Cd) stress has been widely studied over the last decades [1,2]. Thousands of experiments with various species proposed many hypotheses regarding the Cd tolerance mechanisms and Cd toxic action in plants. The main complications in unification of plant tolerance mechanisms to Cd are various time of exposure and applied concentrations. Sanità Di Toppi and Gabbrielli [1] pointed out that the major problem of the Cd–plant interaction is the use of a high Cd concentration for a short time, while a common concentration in standard soil is typically less than 1 µM. This implies that most of the papers studied acute stress (high concentrations with short-time exposure), which reflects environmental conditions inadequately. Studies dealing with chronic effect of Cd (long-time, low-concentration) in terrestrial [3,4] or aquatic plants [5,6] including algae [7] are less frequent. At the same time, exposure to low concentrations of metals including Cd may evoke the so-called hormetic effect. 

Cadmium (Cd) is readily absorbed by plants but has no known physiological function. Members of the Asteraceae family (such as chamomile and dandelion) also readily absorb considerable amounts of Cd in the shoots but also exhibit various metabolic responses [8,9]. Among them, changes in non-enzymatic antioxidants and chelators such as ascorbic acid, thiols, organic acids, and phenols are the most common not only in Cd-exposed plants [10] but also under abiotic stress more generally [11]. 

Plants belonging to the Carlina genus (Asteraceae family) contain various biologically active compounds [12,13]. Hence, they have been widely used in folk medicine [14]. Moreover, some species of Carlina, including *Carlina aculis* L., are facultative metallophytes, i.e., species that tolerate soils with elevated content of heavy metals. Two species, *C. acaulis* and *Carlina vulgaris*, are part of the flora of calamine areas located in metalliferous sites in Bolesław in Southern Poland [15,16].

The aim of this study was to investigate similarities and differences in the responses of *C. acaulis* exposed to low Cd concentrations over a long time (chronic stress, ChS) or to high Cd concentrations over a short time (acute stress, AS). The expected eventual hormetic effect of low Cd dose was studied at the level of various metabolites and data are precisely compared with related (Asteraceae family) or unrelated (metal hyper/accumulators) species.

## 2. Results and Discussion

### 2.1. Impact of Chronic/Acute Cd Stress on the Growth

**Figure 1:**
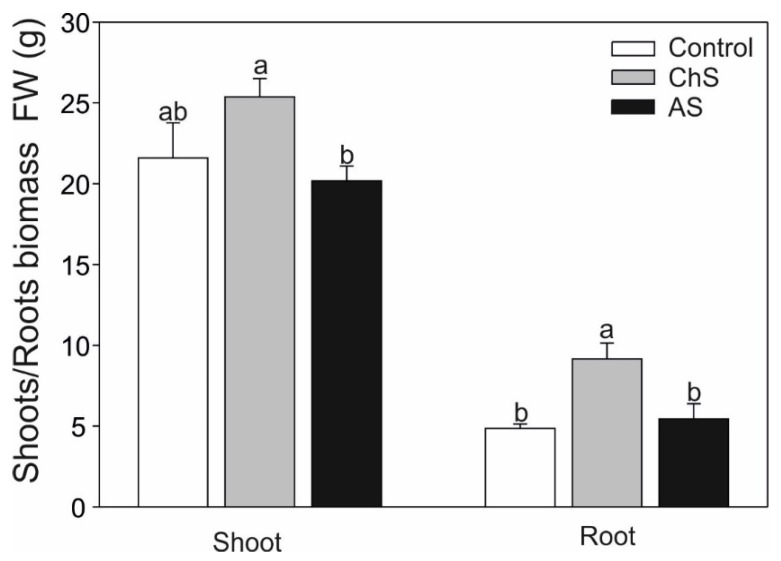
Effect of long-term chronic cadmium stress (ChS, 0.1 µM Cd, 85 days) or short-term acute cadmium stress (AS, 10 µM Cd, 4 days) on the fresh weight of *Carlina acaulis* organs. Data are means ± SE (*n* = 8–10); values followed by the same letter are not significantly different (*p* < 0.05, Tukey’s test).

Cadmium induces numerous changes in plants, including reduction of growth, modified morphology, chlorosis/necrosis, etc. [1,17]. Larsson et al. [18] noted that even 0.5 µM of Cd can decrease leaf area while dose above 2.0 µM of Cd can significantly reduce chlorophyll content. In agreement with these findings, the short-term 10 µM Cd stress in the present study resulted in visible necrotic symptoms (Figure S2). Plants cultured with low Cd concentration (0.1 µM) showed significantly higher root biomass (by ca. 80%) in comparison with the control roots and shoot biomass also showed increase (by ca. 20%) but the difference was not significant (Figure 1). The growth stimulatory effect of the low concentrations of non-essential toxic metals is related to the so-called hormetic effect [19]. As indicated by Calabrese [20], hormesis is an adaptive compensatory process in response to stress and initial disruption in homeostasis. One of the mechanisms responsible for stimulation of growth evoked by the low Cd dose could be an increase in cell proliferation by functional substitution of Zn (by Cd), which is a cofactor of enzymes playing a major role in replication and translation [21].

### 2.2. Accumulation of Cd and Mineral. Nutrients

**Figure 2:**
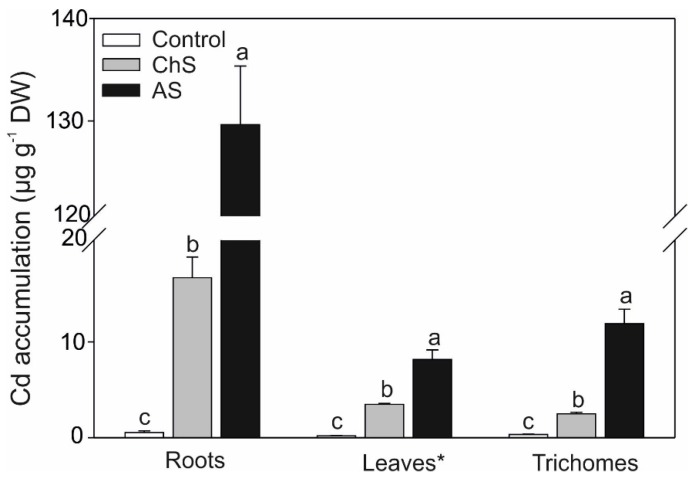
Effect of long-term chronic cadmium stress (ChS, 0.1 µM Cd, 85 days) or short-term acute cadmium stress (AS, 10 µM Cd, 4 days) on the Cd accumulation in the roots, leaves (* leaves without trichomes), and trichomes of *C. acaulis*. Data are means ± SE (*n* = 5); values followed by the same letter are not significantly different (*p* < 0.05, Tukey’s test).

The content of Cd in the plants was significantly affected by its concentration and exposure time (Figure 2). The 10 µM Cd treatment (acute stress) resulted in almost 8-fold higher Cd accumulation in the roots, compared to the 0.1 µM Cd treatment (chronic stress). Accumulation of Cd in the aboveground organs (leaves) was also ca. 2-fold higher in favor of acute stress with even higher differences in trichomes (Figure 2). These data indicate that acute stress evoked higher accumulation of Cd in the Carlina organs probably due to “break” of the protective mechanisms, while plants exposed to chronic stress had time to adapt to the Cd presence by modulation of protective metabolites, as mentioned below. Despite lower absolute Cd content, accumulation efficiency was higher under ChS if expressed as shoot bioaccumulation factor (shoot Cd amount as µg per g DW/solution Cd as µg per mL; see [22] for details) with a value of ca. 280. In the related species chamomile (Asteraceae family), cultivation with 1.5 µM Cd over 7 weeks led to shoot bioaccumulation factor (BAF) of only 145 while Cd/Ni hyperaccumulators from the Thlaspi genus had shoot BAF over 600 or 1300, respectively [22]. With increasing exogenous Cd dose, BAF values typically decrease, e.g., rice exposed to ca. 4.5–18 µM Cd over 42 days had shoot BAF ca. 30–15 [23]. It seems that *C. acaulis* is relatively good accumulator of Cd at lower concentrations and survey of samples collected from nature could show an eventual hazard for human health when used in folk medicine.

Cd is a highly mobile element, and its progressive accumulation in relation to longer exposure time has been observed in various species [3,9]. Owing to greater increase in the root Cd content under AS, the translocation factors (TF) leaf/root or trichome/root decreased and trichome/leaf TF increased (Table S1). This phenomenon has not yet been reported and we assume that under high Cd concentration (acute stress in our work), Cd can partly be detoxified by storage in the leaf trichomes and this mechanism of Cd detoxification has been proposed in several species [24,25,26]. Leaf/root TF under chronic stress was ca. 0.2 (Table S1), which is similar to related species (Asteraceae family) such as chamomile cultured with 1.5 µM Cd over 7 weeks (TF ca. 0.13; [22]), dandelion cultured with 30 µM Cd over 2 weeks (TF ca. 0.19; [9]) or *Tanacetum parthenium* cultured with 35–70 µM Cd over 40 days (TF ca. 0.11–0.20; [27]). Owing to preferential Cd accumulation in the roots, *C. acaulis* is a clear excluder of Cd. 

Several mechanisms of the impact of Cd on the uptake of nutrients have been postulated: competition for Ca transporters, inhibition of Fe loading to the xylem, or indirect influence on nutrient movement [28]. Our results showed that the exposure to both Cd treatments resulted in a decrease in Ca and Mg root accumulation. Additionally, Cd depleted the content of K and Cu in the leaves (Table 1). These results are in agreement with previous studies [29] which showed that two Atriplex species exposed to Cd contained significantly lower levels of K and Ca. Interestingly, it was found that leaves exposed to ChS accumulated more Ca than the control or AS-treated plants. The elevated amount of Ca in the leaves under low Cd stress may be a sign of some protective mechanism. The role of Ca in attenuation of Cd toxicity is known [29,30] and several hypotheses have been put forward, including antioxidative protection, competition of Ca for the same channel transporters with Cd, improved antioxidant enzyme activities [31], or even excretion of Ca-Cd crystals through trichomes [25]. Micronutrients (such as Cu or Zn) were rather negatively affect by some of the stresses (Table 1) while Mn increased in ChS-exposed plants, indicating a need for further study of Cd–Mn interactions.

### 2.3. Changes in Antioxidants and Chelators Differ. under Acute and Chronic Cd Stress

**Figure 3:**
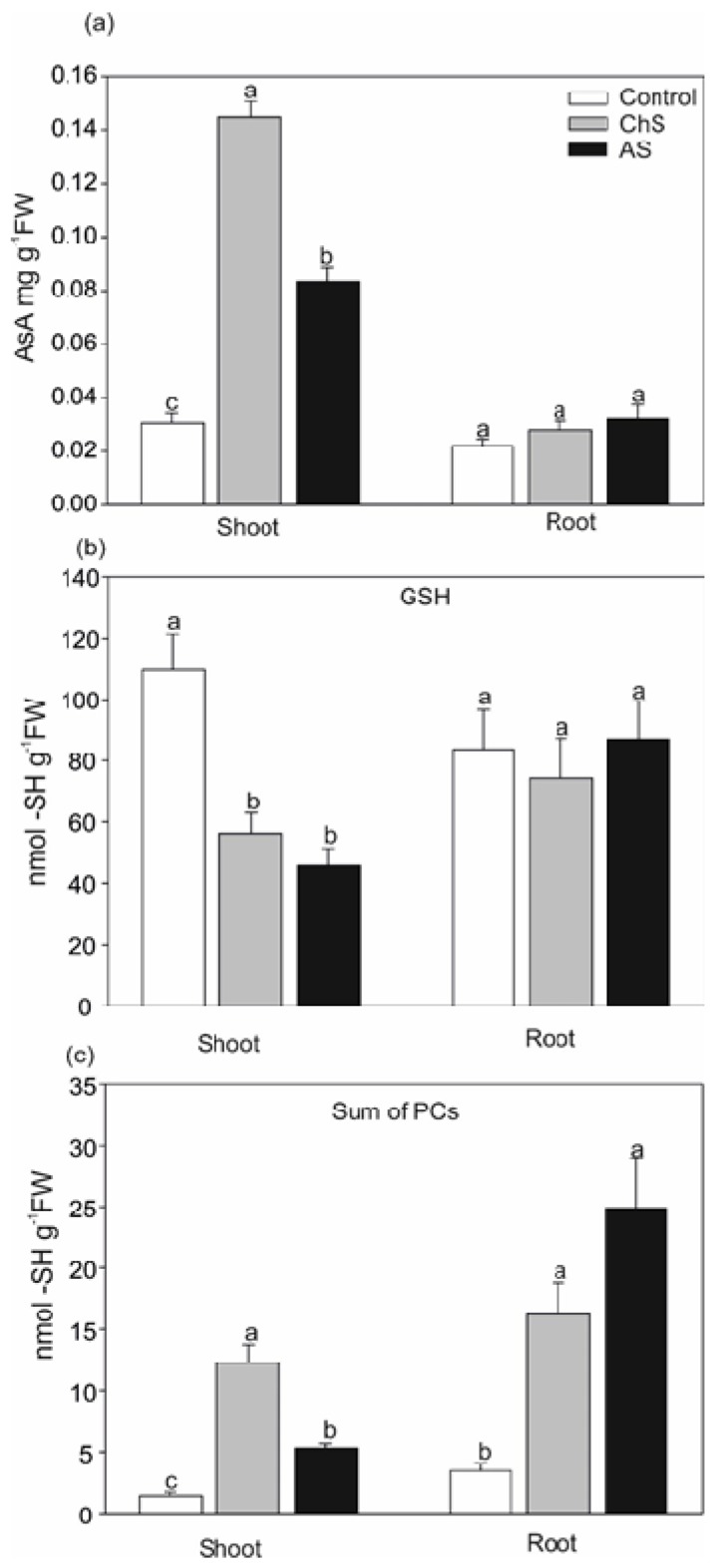
Effect of long-term chronic cadmium stress (ChS, 0.1 µM Cd, 85 days) or short-term acute cadmium stress (AS, 10 µM Cd, 4 days) on (**a**) ascorbic acid (AsA); (**b**) glutathione (GSH); and (**c**) sum of phytochelatins (PCs) content in the shoots and roots of *C. acaulis*. Data are means ± SE (*n* = 5); values followed by the same letter are not significantly different (*p* < 0.05, Tukey’s test).

Both AsA and GSH are essential components of the ascorbate-glutathione pathways of ROS removal. Interestingly, both AS and ChS elevated the AsA amount but reduced the GSH content in the shoots, indicating possible reciprocal changes between these antioxidants (Figure 3a,b). At the same time, the root contents of these metabolites remained unaffected and indicated more pronounced changes in the photosynthetic tissue. In agreement, ascorbic acid is often elevated by Cd excess but GSH rather decreases during long-term exposure [10]. Lower GSH content in the shoots also confirms its role as a precursor for PCs [32,33], while a more intensive increase in PCs without any impact on the GSH content may indicate more intensive biosynthesis of GSH as a protective mechanism against higher accumulation of Cd in the roots (compared to shoots).

An increasing AsA level was determined in the shoots of plants exposed to ChS and AS (Figure 3a). However, the highest accumulation of AsA was observed in the ChS shoots. It has been shown that AsA protects plant cells against Cd-induced oxidative damage [34] and the age of plants a has significant impact on AsA accumulation under Cd stress [35]. 

Contrary to AsA and/or GSH accumulation, the content of the sum of PCs in the shoots differed between the treatments (Figure 3c). PCs are considered as one of the major Cd intercellular chelating ligands and their content is usually related to the metal concentration [36]. In their review paper, Sanità Di Toppi and Gabbrielli [1] pointed out that PCs are the main mechanism of plant cells to cope with Cd stress; however, they indicated that most investigations were focused on acute Cd stress. Our results showed that the Cd stress (both treatments) significantly elevated the sum of PCs, but the ChS plants had over 2-fold higher PC content than in the AS treatment (Figure 3c). The data showed that even at the lower Cd accumulation in the ChS shoots, the content of PCs was higher than in the AS treatment which involved higher doses of Cd (cf. Figure 2; Figure 3c), suggesting that the exposure time, in addition to the applied metal dose, also plays a role. Sun et al. [36] suggested that an increasing Cd concentration in the nutrient medium elevates PC content but Cd concentration above a critical value reduces the content of PCs due to severe toxicity. On the other hand there is evidence that even 20 nM of Cd can induces PC in *Ceratophyllum demersum* [6].

**Figure 4:**
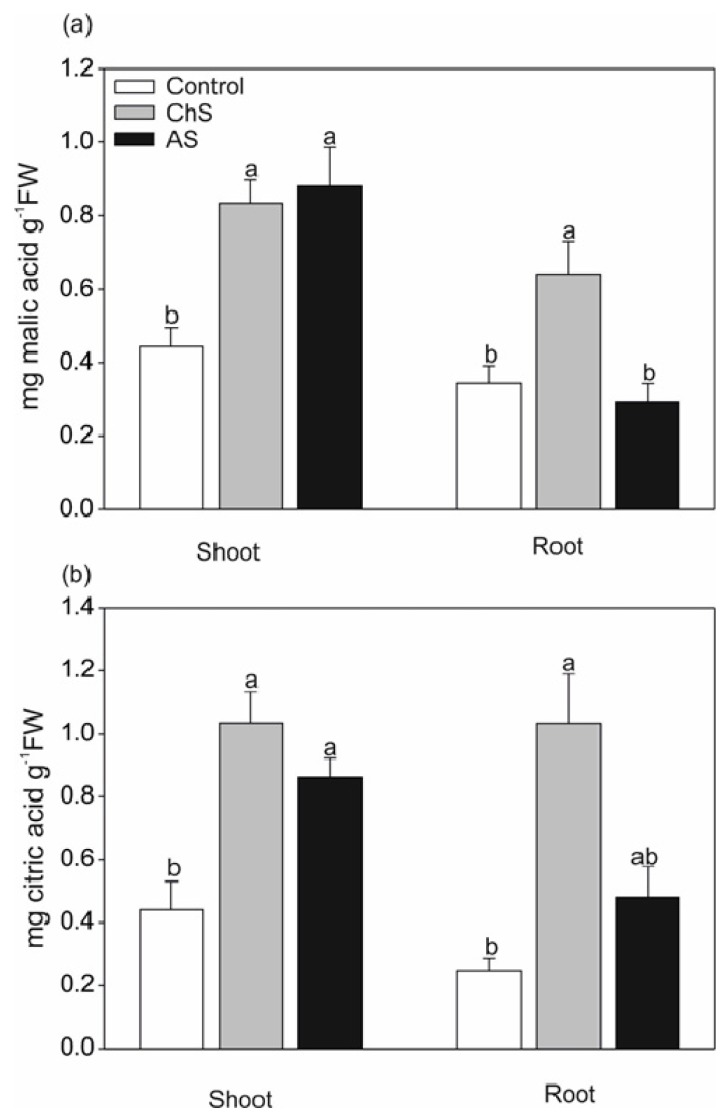
Effect of long-term chronic cadmium stress (ChS, 0.1 µM Cd, 85 days) or short-term acute cadmium stress (AS, 10 µM Cd, 4 days) on (**a**) malic acid; and (**b**) citric acid content in the shoots and roots of *C. acaulis*. Data are means ± SE (*n* = 5); values followed by the same letter are not significantly different (*p* < 0.05, Tukey’s test).

Organic acids are efficient compounds in detoxification of heavy metals. They are involved in several mechanisms including (1) reduction of metal availability by chelation with exudates; (2) intracellular metal chelation; (3) long-distance translocation of metals to compartments with low biological activity such as trichomes and the cell wall [37,38,39]. Organic acids are produced in response to Cd by various species of vascular plants [39,40] or algae [34]. In the present work, accumulation of citric and malic acids significantly increased (ca. by 2–3-fold compared with control) in both ChS and AS shoots but only in the roots in the ChS treatment (Figure 4). It seems that elevated accumulation of organic acids may be a mechanism for tolerance to chronic Cd stress, perhaps through exudation since the ChS treatment resulted in a lower level of Cd in the tissues than in the AS treatment (cf. Figure 2; Figure 4). This phenomenon has been described in various species including crops [41].

### 2.4. Changes of Secondary Metabolites under Various Exposure

**Figure 5:**
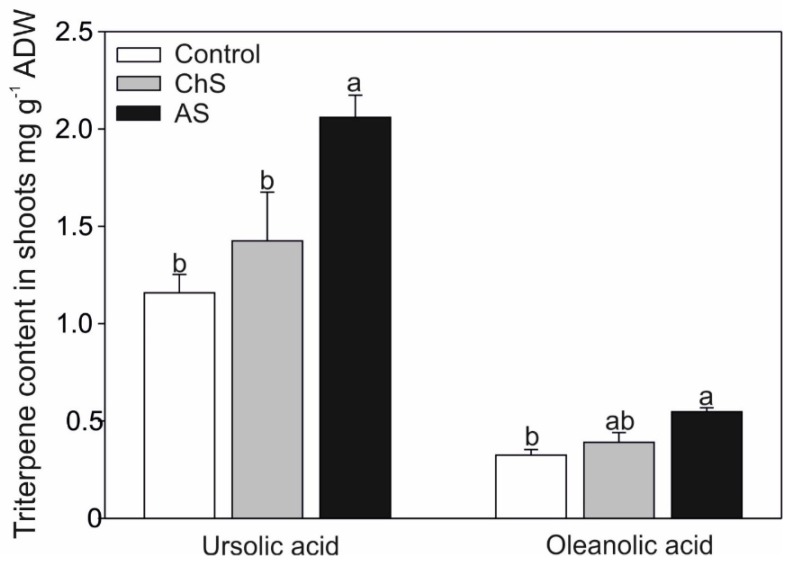
Effect of long-term chronic cadmium stress (ChS, 0.1 µM Cd, 85 days) or short-term acute cadmium stress (AS, 10 µM Cd, 4 days) on ursolic and oleanolic acids content in the shoots of *C. acaulis*. Data are means ± SE (*n* = 5); values followed by the same letter are not significantly different (*p* < 0.05, Tukey’s test).

Two triterpene acids were detected in the shoots only and their accumulation was significantly enhanced (approx. 170% of control) in the AS treatment (Figure 5). The concentration of these acids in response to heavy metals has only rarely been discussed in the literature, mainly in relation to antioxidant properties, which may also play a role under metal stress [42]. It has been shown that heavy metal stress increased ursolic acid in *Prunella vulgaris* [43] and Cd elicited higher accumulation of oleanolic acid in the cell cultures of *Achyranthes bidentate* [44] or triterpenoid saponins in *Bacopa monnieri* [45]. However, a negative effect of Cd or Cu stress on the content of triterpene has also been observed in plant cultures of *Centella asiatica* [46]. Wang et al. [44] suggested that induction of oleanolic acid accumulation is related to the exposure time to Cd and probably to gene expression of 3-hydroxy-3-methylglutaryl coenzyme A reductase in this pathway. 

**Figure 6:**
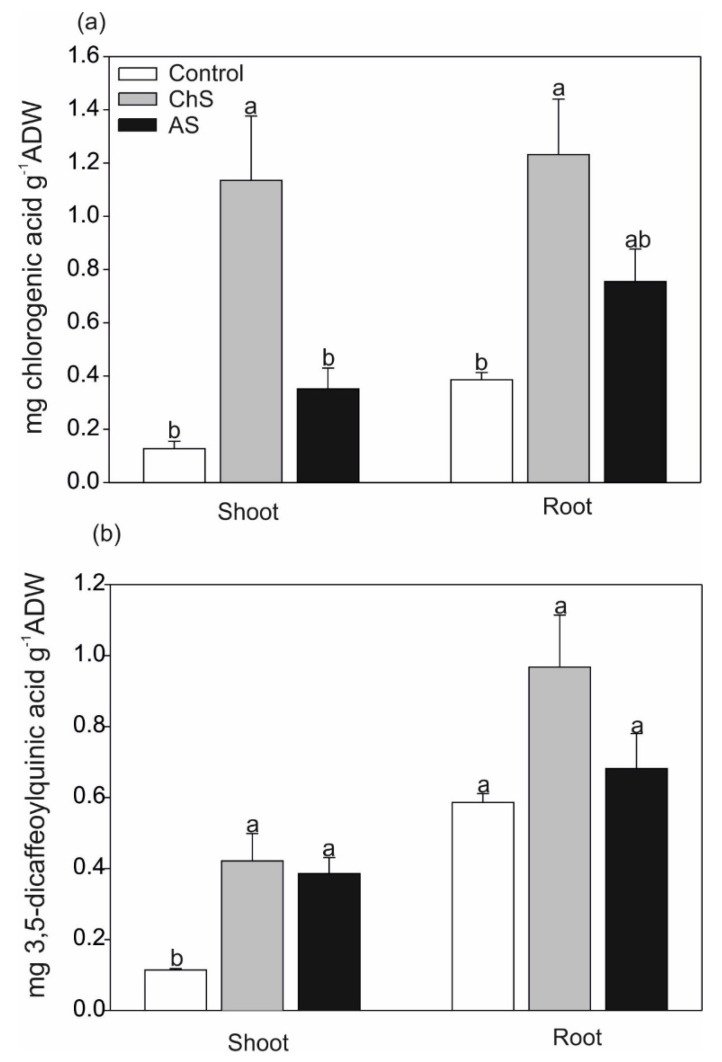
Effect of long-term chronic cadmium stress (ChS, 0.1 µM Cd, 85 days) or short-term acute cadmium stress (AS, 10 µM Cd, 4 days) on (**a**) chlorogenic acid and (**b**) 3,5-dicaffeoylquinic acid in the shoots and roots of *C. acaulis*. Data are means ± SE (*n* = 5); values followed by the same letter are not significantly different (*p* < 0.05, Tukey’s test).

ChS considerably stimulated (over 9.4-fold for shoot and 3.2-fold for root, compared with control) mainly the accumulation of chlorogenic acid in both organs, while the AS treatment induced an increase in the content of this acid which was not significantly affected (Figure 6a). The accumulation of 3,5-dicaffeoylquinic acid was significantly elevated in the shoots only (by approx. 2.5-fold) (Figure 6b). This increase in both phenolic acids under the ChS treatment was related to higher TPC and antioxidant capacity in the roots, compared to the control, and significant elevation of TPC in the shoots compared to AS (Figure S3). In an earlier work, Kováčik and Klejdus [8] observed significant elevation of chlorogenic acid in the related species chamomile (Asteraceae family) after prolonged exposure even to a low Cd dose (3 µM), indicating that chlorogenic acid has probably more general antioxidative action. The negative effect of multi-heavy metal stress on chlorogenic acid accumulation has also been observed in *Carlina vulgaris* plants collected from metalliferous areas [15]: the authors found that plants inhabiting heavy metal polluted areas accumulated less soluble phenolics and flavonoids and exhibited lower antioxidant capacity than plants from non-polluted areas.

### 2.5. Principle Component Analysis

**Figure 7:**
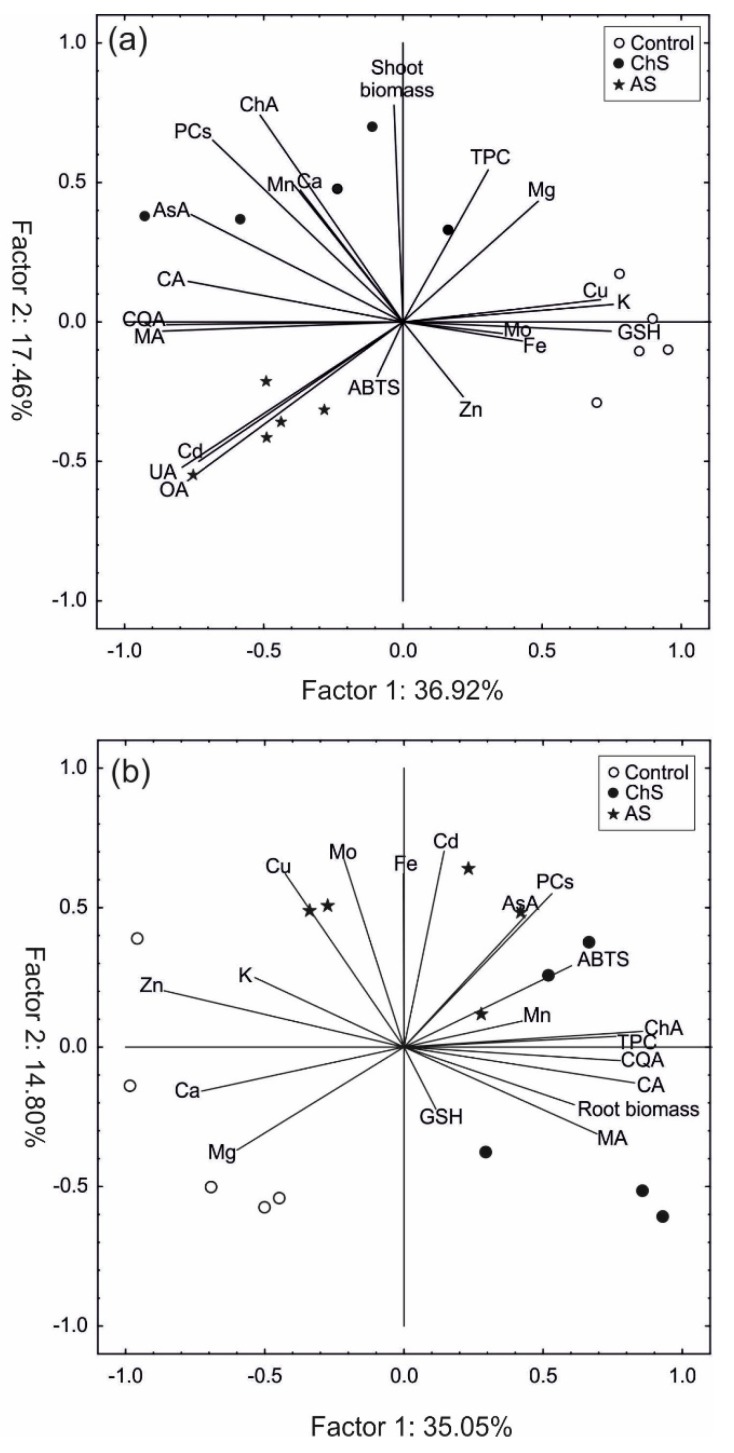
Scaled scatter plot of principal component analysis of selected secondary metabolites (ChA—chlorogenic acid; CQA—3,5-dicaffeoylquinic acid; UA—ursolic acid; OA—oleanolic acid; TPC—total phenolic content), thiol-peptides (GSH—glutathione; PCs—phytochelatins), organic acids (MA—malate acid; CA—citrate acid), antioxidant capacity (ABTS), biomass, macro- and microelements in the shoots (**a**) and roots (**b**). The length of lines shows a correlation between original data and factor axes.

The PCA of the obtained variables, especially from the shoots (Figure 7a), clearly separated the individuals into three groups according to the experimental treatments. The first factor explained 37% and 35% of total variability for the shoots and roots, respectively, while the second factor explained 17% for the shoots and 15% for the roots (Figure 7a,b). This means that both factors explained approx. 54% and slightly more than 50% of the total variance for the shoots and roots, respectively. In the case of the shoots, the first factor facilitated separation of both Cd-stressed groups of plants from the control, and factor 1 was positively correlated with GSH, K, and Cu and negatively correlated with both organic acids, 3,5-dicaffeoylquinic acid, AsA, PCs, Cd, and triterpenes. On the other hand, the shoot biomass was strongly correlated with factor 2. Factor 2 was also partially determined by the Cd, ursolic, and oleanolic acid variables, whose high contents were noted in the shoots of the AS plants (Figure 7a). In the roots, factor 1 distinguished the control and ChS plants (Figure 7b). The first component was positively determined by the content of phenolic compounds, organic acids, and biomass and negatively correlated with Zn, Ca, and Mg. In turn, factor 2 separates the AS roots–individuals with high Cd, Mo, Fe, Cu, and partially AsA and PC content.

## 3. Materials and Methods 

### 3.1. Plant. Material, Growth Conditions, and Experimental Design

The achenes of *Carlina acaulis* L. (Asteraceae, voucher specimen no. 2005A) were obtained from the Botanical Garden of Maria Curie-Skłodowska University in Lublin. They were germinated on the surface of garden soil and 10-day old seedlings were transferred into polyethylene pots filled with garden soil. After 28 days of soil cultivation, the plants were carefully washed with distilled water and transferred into pots (one plant per pot) with 0.5 L of half strength Hoagland’s hydroponic medium [40]. After 5 days of acclimation, the plants were divided into three groups (15 plants per treatment): (1) control plants (continual hydroponic cultivation without addition of Cd), (2) chronic stress/ChS (85 days of cultivation with 0.1 µM Cd), and (3) acute stress/AS (4 days of exposure to 10 µM Cd): total cultivation in hydroponics was 90 days in all treatments (see time axes in Figure S1). The culture solutions were continuously aerated, evapotranspiration loss was replenished daily with dH_2_O, and the medium was renewed every 14 days to prevent micronutrient deficiency. Cadmium was added in the form of Cd(NO_3_)_2_·4H_2_O (Sigma-Aldrich, St. Louis, MO, USA). Cultivation was carried out in a growth chamber at 18/25 °C (night/day) under light-emitting diodes at photosynthetic photon flux density of 90 µmol m^−2^ s^−1^ and relative humidity of 60–65%. Plants from all treatments were harvested after total 90 days of cultivation in hydroponics. Plants were separated into shoots and roots and the fresh weight was determined. Five individual plants were powdered in liquid nitrogen and stored at −80 °C for determination of organic acids, phytochelatins (PCs), glutathione (GSH), and ascorbic acid (AsA). Parallel aliquots were dried at room temperature for determination of secondary metabolites and at 70 °C to constant weight for quantification of Cd and mineral nutrients. Two parallel repetitions of the whole experiments were performed to verify the responses of the main parameters including growth changes, Cd accumulation, and thiol content.

### 3.2. Determination of Cd and Mineral. Nutrients

Cd and mineral nutrients were determined in trichomes and parenchyma separately. The separation of trichomes from parenchyma was achieved in several steps. First, dry shoots were milled, then the trichomes were separated from parenchyma by shaking (WL-2000 shaker, JWElectronic, Warsaw, Poland) on a perforated plate with pore size 1.5 mm. These processes were repeated until clean trichomes fraction was obtained without visible parenchyma particles. The roots, trichomes, and leaves without trichomes were digested in 5 mL of a mixture of HNO_3_:H_2_O (2:8 *v*/*v*) in a microwave digestion apparatus (TOPwave, Analytick Jena AG, Jena, Germany). The resulting clear solutions were transferred into volumetric flasks and filled up to 25 mL with deionized water. The amounts of all elements were measured using ICP-OES PlasmaQuant PQ 9000 Elite (Analityk Jena AG, Jena, Germany). Effective plasma power was 1300 W and the plasma, auxiliary, and nebulizer argon flow rate were 12.0, 0.5, and 0.6 L/min, respectively. Attenuated axial direction of measurement for Ca, K, Mg and axial direction for Cd, Zn, Mn, Fe, Mo, Cu were applied. Each sample was measured in three replicates.

### 3.3. Measurement of Organic Acids, Ascorbic Acid, and Thiols

The organic acids, AsA, and thiols were analyzed in the plant tissue using Agilent 7100 Capillary Electrophoresis (Agilent Technologies, Santa Clara, CA, USA): the organic acids (malic and citric acids) according to the method proposed by [40], total AsA content following [35], and thiols (phytochelatins and glutathione) after monobromobimane derivatization [33]. 

### 3.4. HPLC of Triterpene and Phenolic Acids 

A total of 0.5 g of dried plant material was extracted three times with 100% methanol (3 × 1.5 mL) in an ultrasonic bath for 30 min. The extracts were combined, filtered through 0.22 nylon filters, and filled up to 5 mL in a volumetric flask. High performance liquid chromatography analyses were performed on a VWR Hitachi Chromaster 600 chromatograph (Merck, Darmstadt, Germany) with a PDA detector (Merck, Darmstadt, Germany) and EZChrom Elite software (ver. 3.18, Merck, Darmstadt, Germany). The RP18e LiChrosper 100 column (Merck, Darmstadt, Germany) (25 cm × 4.9 mm i.d., 5 µm particle size) was used to separate triterpenic acids—oleanolic and ursolic acid. Other technical details are the same as reported previously [12]. Chlorogenic and 3,5-dicaffeoylquinic acids were analyzed using C18 reversed-phase column Kinetex (Phenomenex, Torrance, CA, USA) (10 cm × 4.0 mm i.d., 2,6 µm particle size) according to previous work [47]. The identity of compounds was confirmed by comparison of the retention time and spectral similarity with standards. 

### 3.5. Quantification of Total Phenolic Content and Antioxidant Capacity

Analyses were performed in the same methanolic extract used for determination of triterpene and phenolic acids. The total (soluble) phenolic content (TPC) expressed as mg of gallic acid equivalents per gram of air dry weight of plants was measured using the Folin–Ciocalteu reagent as described previously [9]. The antioxidant capacities expressed as mg of trolox equivalents per gram of air dry weight were measured using free radical ABTS/2-azino-bis-3ethyl-benzthiazoline-6 sulphonic acid [48].

### 3.6. Statistical Analysis

Samples from five individual plants were assessed for each treatment, parameter, and organ (*n* = 5). One-way ANOVA followed by a Tukey’s post-hoc test was used to evaluate the significance of differences (*p* < 0.05) between treatments. Principal component analyses (PCA) were performed separately for shoots and roots based on all studied parameters. All statistical analyses were carried out using Statistic ver. 13.3 software (TIBCO Software Inc. 2017, Palo Alto, CA, USA).

## 4. Conclusions

The present study demonstrated some different physiological responses of *Carlina acaulis* to chronic (long time/low concentration) and acute (short time/high concentration) Cd stress. Although mineral nutrients were typically negatively affected by Cd, chronic stress had fewer negative effects and even stimulated root growth, probably due to lower endogenous accumulation of Cd. At the same time, ascorbic acid and phytochelatins were more elevated in the shoots but the content of organic (malic and citric) acids increased in the roots of plants from chronic treatment. In combination with the strongly elevated chlorogenic acid content in this treatment, both chelation and enhancement of antioxidative power are expected to contribute to protection in the plants exposed to the long-term Cd stress. On the contrary, the role of triterpene acids in chronic or acute stress tolerance was not immediately apparent. 

## Figures and Tables

**Table 1:** Accumulation of selected mineral nutrients in the leaves (without trichomes), roots, and trichomes of *C. acaulis* exposed to long-term chronic cadmium stress (ChS, 0.1 µM Cd, 85 days) or short-term acute cadmium stress (AS, 10 µM Cd, 4 days). Data are means ± SE (*n* = 5); values followed by the same letter are not significantly different between treatments (*p* < 0.05, Tukey’s test).

	**Ca mg**·**g^−1^ DW**			**Mg mg**·**g^−1^ DW**		
	**leaf**	**root**	**trichomes**	**leaf**	**root**	**trichomes**
**Control**	13.1 ± 0.3 b	5.81 ± 0.37 a	11.7 ± 0.9 a	5.53 ± 0.15 a	4.31 ± 0.92 a	2.78 ± 0.15 a
**ChS**	14.6 ± 0.3 a	4.16 ± 0.16 b	13.6 ± 0.5 a	5.58 ± 0.35 a	2.44 ± 0.21 b	3.07 ± 0.23 a
**AS**	13.7 ± 0.4 b	4.12 ± 0.48 b	13.7 ± 0.7 a	4.96 ± 0.22 a	2.41 ± 0.11 b	3.04 ± 0.09 a
	**K mg**·**g^−1^ DW**			**Fe µg**·**g^−1^ DW**		
	**leaf**	**root**	**trichomes**	**leaf**	**root**	**trichomes**
**Control**	85.0 ± 3.0 a	46.5 ± 3.2 a	28.6 ± 2.0 a	79.9 ± 3.3 a	3153 ± 682 a	129.8 ± 6.3 a
**ChS**	74.8 ± 4.4 b	42.4 ± 1.8 a	29.7 ± 3.7 a	72.8 ± 6.7 a	3535 ± 517 a	116.8 ± 17.6 a
**AS**	70.9 ± 1.9 b	45.6 ± 3.9 a	28.2 ± 1.2 a	76.8 ± 3.4 a	3463 ± 325 a	128.5 ± 6.4 a
	**Cu µg**·**g^−1^ DW**			**Mn µg**·**g^−1^ DW**		
	**leaf**	**root**	**trichomes**	**leaf**	**root**	**trichomes**
**Control**	6.13 ± 0.34 a	12.1 ± 1.3 a	6.71 ± 0.85 a	7.02 ± 0.72 b	5.80 ± 0.86 b	7.06 ± 1.40 a
**ChS**	4.82 ± 0.37 b	10.6 ± 1.9 a	4.91 ± 0.47 a	9.92 ± 1.15 a	10.10 ± 0.92 a	7.65 ± 1.42 a
**AS**	4.56 ± 0.53 b	11.8 ± 1.3 a	5.35 ± 0.49 a	7.35 ± 1.03 b	9.13 ± 1.32 a	6.96 ± 0.68 a
	**Zn µg**·**g^−1^ DW**			**Mo µg**·**g^−1^ DW**		
	**leaf**	**root**	**trichomes**	**leaf**	**root**	**trichomes**
**Control**	22.4 ± 2.2 a	64.0 ± 2.9 a	25.2 ± 2.8 a	4.45 ± 0.91 a	11.51 ± 4.00 a	2.60 ± 0.48 a
**ChS**	20.1 ± 0.8 a	28.1 ± 1.7 b	17.6 ± 1.9 b	3.09 ± 0.52 a	8.79 ± 4.03 a	2.58 ± 0.39 a
**AS**	21.5 ± 1.8 a	53.0 ± 6.6 a	22.0 ± 3.1 ab	2.94 ± 0.38 a	11.26 ± 1.79 a	2.39 ± 0.21 a

## Supplementary Materials

Supplementary materials can be found at https://www.mdpi.com/1422-0067/21/8/2828/s1.

## References

[1] Sanità Di Toppi L., Gabbrielli R. (1999). Response to cadmium in higher plants. Environ. Exp. Bot..

[2] Clemens S. (2001). Molecular mechanisms of plant metal tolerance and homeostasis. Planta.

[3] Arduini I., Masoni A., Mariotti M., Ercoli L. (2004). Low cadmium application increase miscanthus growth and cadmium translocation. Environ. Exp. Bot..

[4] Kováčik J., Klejdus B., Štork F., Hedbavny J. (2012). Physiological responses of *Tillandsia albida* (Bromeliaceae) to long-term foliar metal application. J. Hazard. Mater..

[5] Andresen E., Kappel S., Stärk H.-J., Riegger U., Borovec J., Mattusch J., Heinz A., Schmelzer C.E.H., Matoušková Š., Dickinson B. (2016). Cadmium toxicity investigated at the physiological and biophysical levels under environmentally relevant conditions using the aquatic model plant *Ceratophyllum demersum*. New Phytol..

[6] Andresen E., Mattusch J., Wellenreuther G., Thomas G., Abad U.A., Küpper H. (2013). Different strategies of cadmium detoxification in the submerged macrophyte *Ceratophyllum demersum* L.. Metallomics.

[7] Perreault F., Dionne J., Didur O., Juneau P., Popovic R. (2011). Effect of cadmium on photosystem II activity in *Chlamydomonas reinhardtii*: Alteration of O–J–I–P fluorescence transients indicating the change of apparent activation energies within photosystem II. Photosynth. Res..

[8] Kováčik J., Klejdus B. (2008). Dynamics of phenolic acids and lignin accumulation in metal-treated *Matricaria chamomilla* roots. Plant. Cell Rep..

[9] Kováčik J., Bujdoš M., Ketzer P., Babula P., Peterková V., Krenn L. (2019). Dandelion is more tolerant to cadmium than to nickel excess. Chemosphere.

[10] Kováčik J., Hasanuzzaman M., Vara Prasad M.N., Nahar K. (2019). Role of low molecular weight compounds in cadmium stress tolerance. Cadmium Tolerance in Plants.

[11] Chrysargyris A., Papakyriakou E., Petropoulos S.A., Tzortzakis N. (2019). The combined and single effect of salinity and copper stress on growth and quality of *Mentha spicata* plants. J. Hazard. Mater..

[12] Strzemski M., Wójciak-Kosior M., Sowa I., Rutkowska E., Szwerc W., Kocjan R., Latalski M. (2016). Carlina species as a new source of bioactive pentacyclic triterpenes. Ind. Crops Prod..

[13] Strzemski M., Wójciak-Kosior M., Sowa I., Agacka-Mołdoch M., Drączkowski P., Matosiuk D., Kurach Ł., Kocjan R., Dresler S. (2017). Application of Raman spectroscopy for direct analysis of *Carlina acanthifolia* subsp. utzka root essential oil. Talanta.

[14] Strzemski M., Wójciak-Kosior M., Sowa I., Załuski D., Verpoorte R. (2019). Historical and traditional medical applications of *Carlina acaulis* L.—A critical ethnopharmacological review. J. Ethnopharmacol..

[15] Strzemski M., Wójciak-Kosior M., Sowa I., Załuski D., Szwerc W., Sawicki J., Kocjan R., Feldo M., Dresler S. (2017). *Carlina vulgaris* L. as a source of phytochemicals with antioxidant activity. Oxid. Med. Cell. Longev..

[16] Jędrzejczyk-Korycińska M. (2006). Floristic diversity in calamine areas of the Silesia-Cracow Monocline. Biodiv. Res. Conserv..

[17] Gallego S.M., Pena L.B., Barcia R.A., Azpilicueta C.E., Iannone M.F., Rosales E.P., Zawoznik M.S., Groppa M.D., Benavides M.P. (2012). Unravelling cadmium toxicity and tolerance in plants: Insight into regulatory mechanisms. Environ. Exp. Bot..

[18] Larsson E.H., Bornman J.F., Asp H. (1998). Influence of UV-B radiation and Cd^2+^ on chlorophyll fluorescence, growth and nutrient content in *Brassica napus*. J. Exp. Bot..

[19] Poschenrieder C., Cabot C., Martos S., Gallego B., Barceló J. (2013). Do toxic ions induce hormesis in plants?. Plant. Sci..

[20] Calabrese E.J. (1999). Evidence that hormesis represents an “overcompensation” response to a disruption in homeostasis. Ecotoxicol. Environ. Saf..

[21] Aina R., Labra M., Fumagalli P., Vannini C., Marsoni M., Cucchi U., Bracale M., Sgorbati S., Citterio S. (2007). Thiol-peptide level and proteomic changes in response to cadmium toxicity in *Oryza sativa* L. roots. Environ. Exp. Bot..

[22] Kováčik J. (2013). Hyperaccumulation of cadmium in *Matricaria chamomilla*: A never-ending story?. Acta Physiol. Plant..

[23] Yu H., Guo J., Li Q., Zhang X., Huang H., Huang F., Yang A., Li T. (2020). Characteristics of cadmium immobilization in the cell wall of root in a cadmium-safe rice line (*Oryza sativa* L.). Chemosphere.

[24] Salt D.E., Prince R.C., Pickering I.J., Raskin I. (1995). Mechanisms of cadmium mobility and accumulation in indian mustard. Plant. Physiol..

[25] Choi Y.-E., Harada E., Wada M., Tsuboi H., Morita Y., Kusano T., Sano H. (2001). Detoxification of cadmium in tobacco plants: Formation and active excretion of crystals containing cadmium and calcium through trichomes. Planta.

[26] Isaure M.-P., Fayard B., Sarret G., Pairis S., Bourguignon J. (2006). Localization and chemical forms of cadmium in plant samples by combining analytical electron microscopy and X-ray spectromicroscopy. Spectrochim. Acta Part. B At. Spectrosc..

[27] Hojati M., Modarres-Sanavy S.A.M., Enferadi S.T., Majdi M., Ghanati F., Farzadfar S., Pazoki A. (2017). Cadmium and copper induced changes in growth, oxidative metabolism and terpenoids of *Tanacetum parthenium*. Environ. Sci. Pollut. Res..

[28] Matraszek R., Hawrylak-Nowak B., Chwil S., Chwil M. (2016). Interaction between cadmium stress and sulphur nutrition level on macronutrient status of *Sinapis alba* L.. Water Air Soil Pollut..

[29] Nedjimi B., Hasanuzzaman M., Fujita M., Oku H., Nahar K., Hawrylak-Nowak B. (2018). Heavy metal tolerance in two Algerian saltbushes: A review on plant responses to cadmium and role of calcium in its mitigation. Plant Nutrients and Abiotic Stress Tolerance.

[30] Kováčik J., Dresler S. (2018). Calcium availability but not its content modulates metal toxicity in *Scenedesmus quadricauda*. Ecotoxicol. Environ. Saf..

[31] Wang C.Q., Song H. (2009). Calcium protects *Trifolium repens* L. seedlings against cadmium stress. Plant. Cell Rep..

[32] Zenk M.H. (1996). Heavy metal detoxification in higher plants-A review. Gene.

[33] Dresler S., Hawrylak-Nowak B., Kováčik J., Pochwatka M., Hanaka A., Strzemski M., Sowa I., Wójciak-Kosior M. (2019). Allantoin attenuates cadmium-induced toxicity in cucumber plants. Ecotoxicol. Environ. Saf..

[34] Kováčik J., Klejdus B., Babula P., Hedbavny J. (2017). Ascorbic acid affects short-term response of *Scenedesmus quadricauda* to cadmium excess. Algal Res..

[35] Dresler S., Maksymiec W. (2013). Capillary zone electrophoresis for determination of reduced and oxidised ascorbate and glutathione in roots and leaf segments of *Zea mays* plants exposed to Cd and Cu. Acta Sci. Pol. Hortorum Cultus.

[36] Sun Q., Ye Z.H., Wang X.R., Wong M.H. (2007). Cadmium hyperaccumulation leads to an increase of glutathione rather than phytochelatins in the cadmium hyperaccumulator *Sedum alfredii*. J. Plant. Physiol..

[37] Kutrowska A., Szelag M. (2014). Low-molecular weight organic acids and peptides involved in the long-distance transport of trace metals. Acta Physiol. Plant..

[38] Hawrylak-Nowak B., Dresler S., Matraszek R. (2015). Exogenous malic and acetic acids reduce cadmium phytotoxicity and enhance cadmium accumulation in roots of sunflower plants. Plant. Physiol. Biochem..

[39] Dresler S., Wójciak-Kosior M., Sowa I., Stanisławski G., Bany I., Wójcik M. (2017). Effect of short-term Zn/Pb or long-term multi-metal stress on physiological and morphological parameters of metallicolous and nonmetallicolous *Echium vulgare* L. populations. Plant. Physiol. Biochem..

[40] Dresler S., Hanaka A., Bednarek W., Maksymiec W. (2014). Accumulation of low-molecular-weight organic acids in roots and leaf segments of *Zea mays* plants treated with cadmium and copper. Acta Physiol. Plant..

[41] Fu H., Yu H., Li T., Zhang X. (2018). Influence of cadmium stress on root exudates of high cadmium accumulating rice line (*Oryza sativa* L.). Ecotoxicol. Environ. Saf..

[42] Do Nascimento P.G.G., Lemos T.L.G., Bizerra A.M.C., Arriaga Â.M.C., Ferreira D.A., Santiago G.M.P., Braz-Filho R., Costa J.G.M. (2014). Antibacterial and antioxidant activities of ursolic acid and derivatives. Molecules.

[43] Wu Z., Guo Q., Wang Q., Zhou L., Zhang Z., Zhang L., Huang T. (2010). Effects of lead, copper and cadmium stresses on growth and inherent quality of *Prunalla vulgaris*. Zhongguo Zhongyao Zazhi.

[44] Wang Q.J., Lei X.Y., Zheng L.P., Wang J.W. (2017). Molecular characterization of an elicitor-responsive 3-hydroxy-3-methylglutaryl coenzyme A reductase gene involved in oleanolic acid production in cell cultures of *Achyranthes bidentata*. Plant. Growth Regul..

[45] Gupta P., Khatoon S., Tandon P.K., Rai V. (2014). Effect of cadmium on growth, bacoside A, and bacopaside I of *Bacopa monnieri* (L.), a Memory Enhancing Herb. Sci. World J..

[46] Kim O.T., Kim M.Y., Hong M.H., Ahn J.C., Hwang B. (2004). Stimulation of asiaticoside accumulation in the whole plant cultures of *Centella asiatica* (L.) Urban by elicitors. Plant. Cell Rep..

[47] Sowa I., Paduch R., Strzemski M., Zielińska S., Rydzik-Strzemska E., Sawicki J., Kocjan R., Polkowski J., Matkowski A., Latalski M. (2018). Proliferative and antioxidant activity of *Symphytum officinale* root extract. Nat. Prod. Res..

[48] Re R., Pellegrini N., Proteggente A., Pannala A., Yang M., Rice-Evans C. (1999). Antioxidant activity applying an improved ABTS radical cation decolorization assay. Free Radic. Biol. Med..

