# Supplementary material for: Tolerance of Facultative Metallophyte Carlina acaulis to Cadmium Relies on Chelating and Antioxidative Metabolites
Source: Int J Mol Sci. 2020 Apr 18;21(8):2828. doi: 10.3390/ijms21082828 (PMC7215424; doi:10.3390/ijms21082828)
Supplement: Supplementary file 1 [file ijms-21-02828-s001.pdf]

SUPPLEMENTARY MATERIALS

# Tolerance of facultative metallophyte *Carlina acaulis* to cadmium relies on chelating and antioxidative metabolites

**Sławomir Dresler [1]*, Maciej Strzemski [2], Jozef Kováčik [3], Jan Sawicki [2], Michał Staniak [2], Magdalena Wójciak [2], Ireneusz Sowa [2], Barbara Hawrylak-Nowak [4]**

[1] Department of Plant Physiology and Biophysics, Institute of Biological Science, Maria Curie-Skłodowska University, Akademicka 19, 20-033 Lublin, Poland; slawomir.dresler@poczta.umcs.lublin.pl

[2] Department of Analytical Chemistry, Medical University of Lublin, Chodźki 4a, 20-093, Lublin, Poland; maciej.strzemski@poczta.onet.pl (M.S.); jan.sawicki@mgr.farm (J.S.); michal_staniak@wp.pl (M.S.); kosiorma@wp.pl (M.W.-K.); i.sowa@umlub.pl (I.S.);

[3] Department of Biology, University of Trnava, Priemyselná 4, 918 43 Trnava, Slovak Republic; jozkovacik@yahoo.com

[4] Department of Botany and Plant Physiology, University of Life Sciences in Lublin, Akademicka 15, 20-950 Lublin, Poland; barbara.nowak@up.lublin.pl

*Correspondence: slawomir.dresler@poczta.umcs.lublin.pl Tel.:+48-81-537-5078

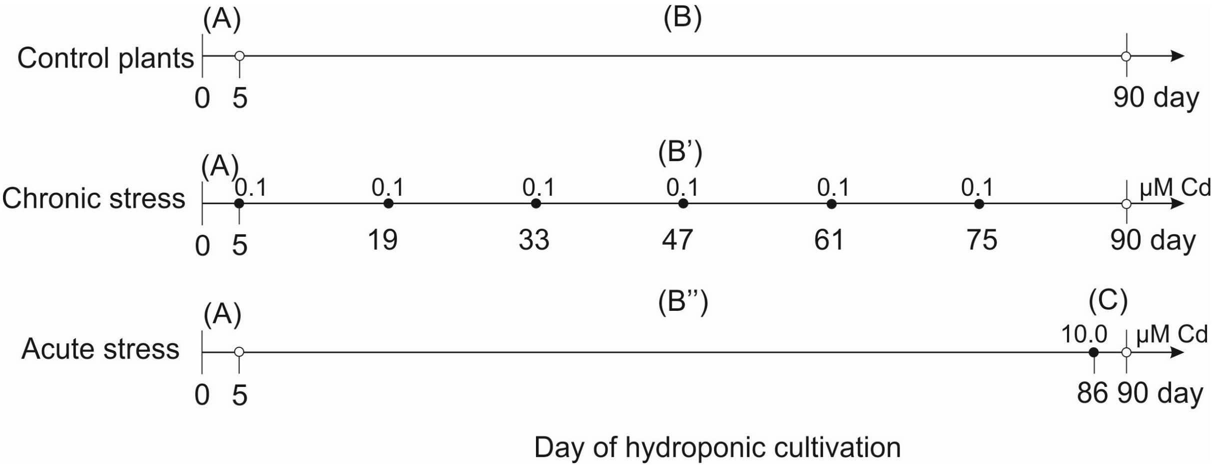


**Suppl. Fig. S1.** Diagram of the experimental design: (A) 5 days of acclimation to hydroponics; (B) 85 days of cultivation in control conditions; (B′) 85 days of cultivation with 0.1 μM Cd = chronic stress; (B′′) 81 days of cultivation without Cd, followed by (C) 4 days of cultivation with 10 μM Cd = acute stress. Total cultivation in hydroponics was 90 days in all treatments. Numbers below axis indicate days when culture solutions were renewed.

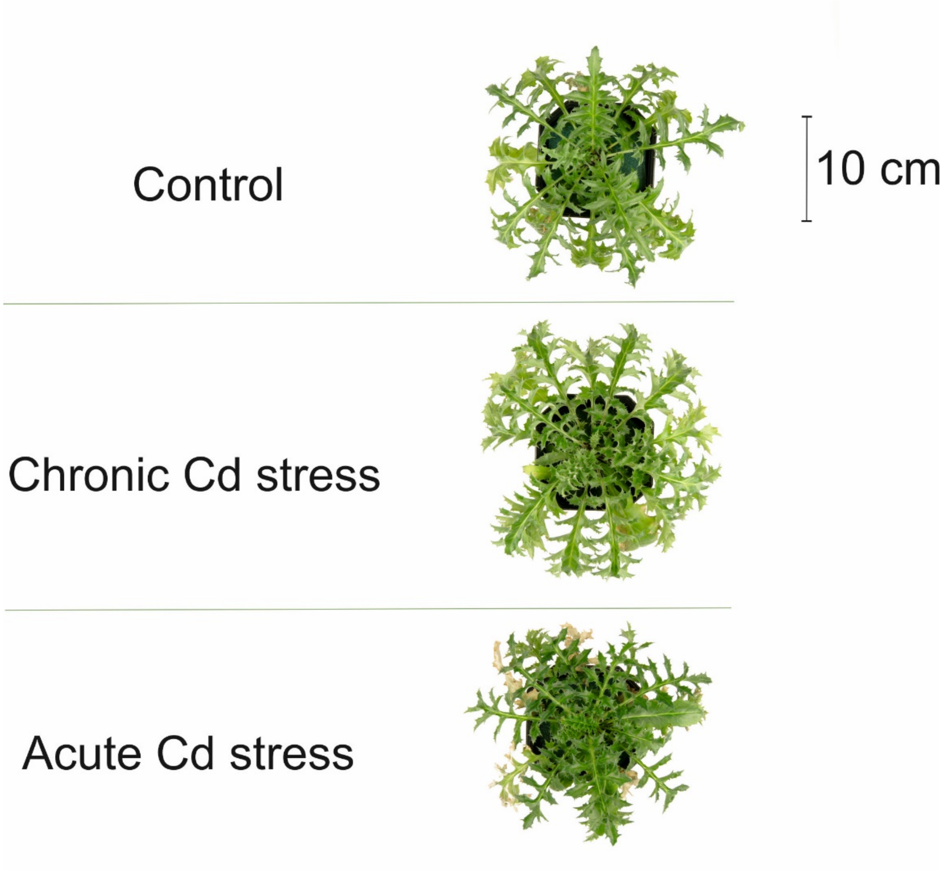


**Suppl. Fig. S2.** Phenotype of *Carlina acaulis* plants at the end of the treatments with various Cd doses and exposure time.

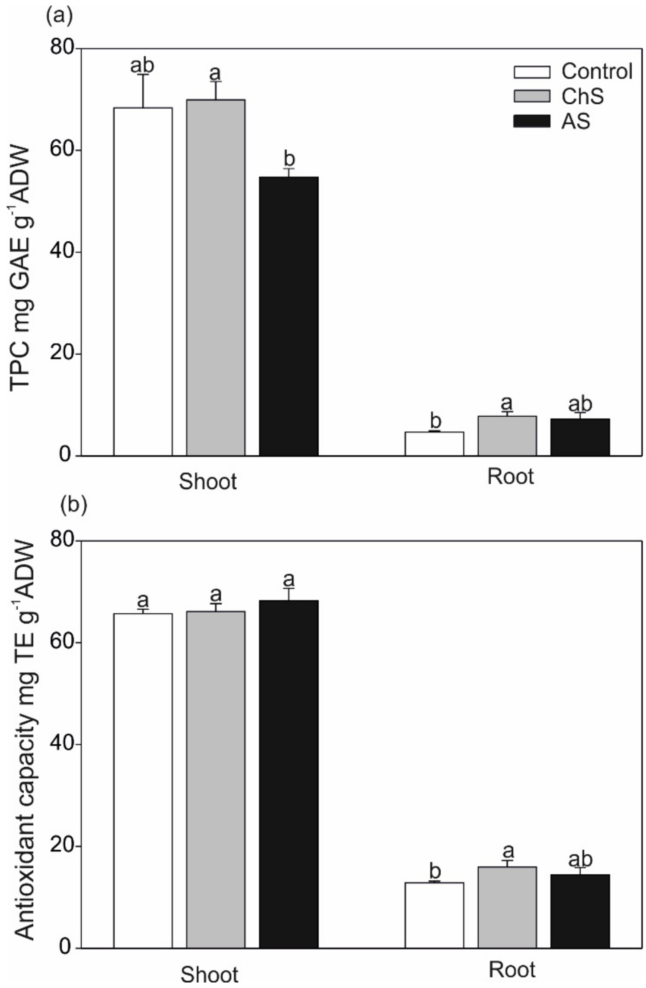


**Suppl. Fig. S3.** Effect of long-term chronic cadmium stress (ChS, 0.1 μM Cd, 85 days) or short-term acute cadmium stress (AS, 10 μM Cd, 4 days) on: (a) total phenolic content (TPC) and (b) antioxidant capacity in the shoots and roots of *C. acaulis*. Data are means ± SE (n=5); values followed by the same letter are not significantly different ($p < 0.05$, Tukey's test).

**Suppl. Table S1.** Translocation factors of Cd in plants exposed to long-term chronic cadmium stress (ChS, 0.1 μM Cd, 85 days) or short-term acute cadmium stress (AS, 10 μM Cd, 4 days).

| | **Leaf/Root** | **Trichome/Leaf** | **Trichome/Root** |
|---|---|---|---|
| ChS | 0.215 | 0.715 | 0.150 |
| AS | 0.061 | 1.457 | 0.088 |
